# Supplementary material for: Lipid-Independent Regulation of PLIN5 via IL-6 through the JAK/STAT3 Axis in Hep3B Cells
Source: Int J Mol Sci. 2023 Apr 13;24(8):7219. doi: 10.3390/ijms24087219 (PMC10138877; doi:10.3390/ijms24087219)
Supplement: Supplementary file 1 [file ijms-24-07219-s001.zip › ijms-2254384-supplementary.pdf]

Supplemental Material

Lipid-Independent Regulation of PLIN5 via IL-6 through the JAK/STAT3 Axis in Hep3B Cells

Marinela Krizanac, Paola Berenice Mass Sanchez, Sarah K. Schröder, Ralf Weiskirchen, and Anastasia Asimakopoulos

Table S1. Antibodies used for Western blot analysis*

Primary antibodies					
**Antibody**	**Cat. No.**	**Supplier**	**Size (kDa)**	**Dilution**	**Source**
Akt	#4685	Cell Signaling	60	1:1000	r
CASP3	#9662	Cell Signaling	35	1:1000	r
cl. CASP3	#9664	Cell Signaling	17/19	1:1000	r
cl. PARP	sc-56196	Santa Cruz	90	1:1000	r
ERK1/2	#9101	Cell Signaling	42/44	1:1000	r
GAPDH	sc-32233	Santa Cruz	38	1:1000	m
Histone H3	#4499	Cell Signaling	17	1:2000	r
IL-6R	sc-373708	Santa Cruz	50/80	1:750	m
LCN2	AF1757	R&D Systems	25	1:1000	g
NF-κB	sc-8008	Santa Cruz	65	1:1000	m
pAkt	#4060	Cell Signaling	60	1:1000	r
PARP	#9532	Cell Signaling	116/89	1:1000	r
pERK1/2	#9102	Cell Signaling	42/44	1:1000	r
PLIN5	26951-1-AP	Proteintech	50	1:1000	r
pNF-κB	#3031	Cell Signaling	65	1:1000	r
pSMAD2	#8828	Cell Signaling	52/60	1:1000	r
pSTAT1	#9167	Cell Signaling	84-91	1:1000	r
pSTAT3	#9134	Cell Signaling	84-91	1:1000	r
SMAD2/3	#8685	Cell signaling	52/60	1:1000	r
STAT1	sc-464	Santa Cruz	84-91	1:1000	m
STAT3	sc-8019	Santa Cruz	84-91	1:1000	m
Vinculin	66305-1-Ig	Proteintech	117	1:2000	r
**Secondary antibodies**					
**Antibody**	**Cat. No.**	**Supplier**		**Dilution**	**Source**
goat anti-mouse IgG (H+L), HRP	#31430	Invitrogen	NA	1:10,000	g
mouse anti-goat IgG (H+L), HRP	#31400	Invitrogen	NA	1:10,000	m
goat anti-rabbit IgG (H+L), HRP	#31460	Invitrogen	NA	1:10,000	g

* Abbreviations used are: g, goat; H+L, respective antibody contains 2 heavy chains (H) and 2 light chains (L); HRP, horseradish peroxidase; IgG, immunoglobulin G; m, mouse; NA, not applicable; r, rabbit.

Table S2. Oligonucleotides used for RT-qPCR

Human Gene	Accession No.	Primers
PLIN5	NM_001013706	forward: 5′-gtggccagcagtgtcacggg-3′<br>reverse: 5′-ggagccgaggcgcacaaagt-3′
IL6R	NM_000565.4	forward: 5′-cccctcagcaatgttgtttgt-3′<br>reverse: 5′-ctccgggactgctaactgg-3′
STAT3	NM_139276.3	forward: 5′-tcctgaagctgacccaggta-3′<br>reverse: 5′-ggcaggtcaatggtattgct-3′
GAPDH	NM_002046.7	forward: 5′-agccacatcgctcagacac-3′<br>reverse: 5′-gcccaatacgaccaaatcc-3′

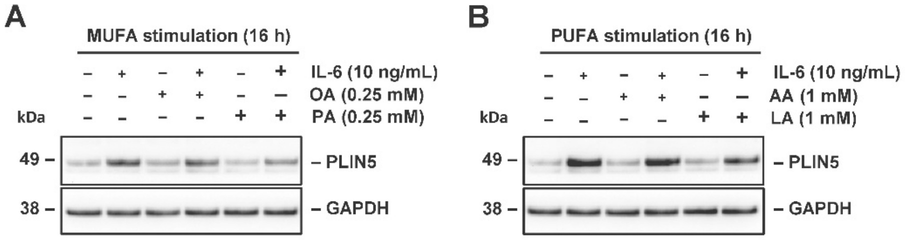

Figure S1. Effects of combined stimulation of IL-6 and fatty acids on PLIN5 expression in Hep3B cells. Hep3B cells were treated with IL-6 in combination with either (**A**) oleic acid (OA) and palmitic acid (PA) or (**B**) arachidonic acid (AA) and linoleic acid (LA) for 16 h. Expression of PLIN5 was analyzed by Western blot using GAPDH to demonstrate equal protein loading. MUFA, monounsaturated fatty acids; PUFA, polyunsaturated fatty acids

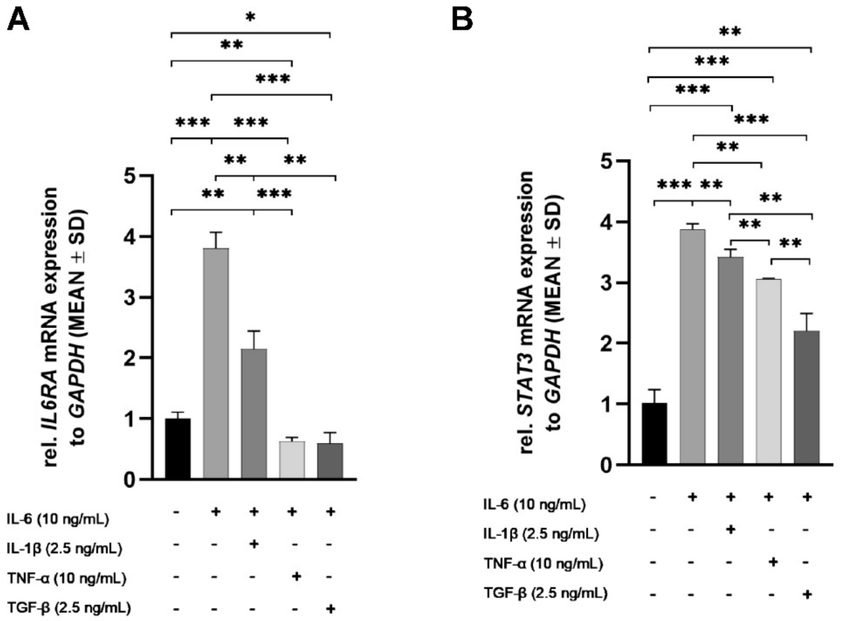

Figure S2. Impact of different cytokines on *IL6RA* and *STAT3* mRNA expression in Hep3B cells. Expression of (**A**) *IL6RA* and (**B**) *STAT3* mRNA in Hep3B cells after stimulation with IL-6, IL-1β, TNF-α, and TGF-β as assessed by RT-qPCR. Untreated cells were used as controls. For RT-qPCR results, data are shown as mean ± SD of 3 independent experiments performed in triplicate and measured in technical replicates. Significant differences between the groups in (**A**) or (**B**) are marked by asterisks (*$p < 0.05$, **$p < 0.01$, ***$p < 0.001$).

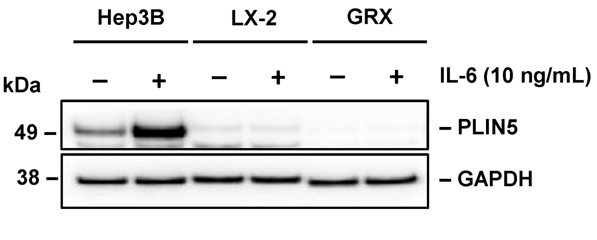

Figure S3. Treatment of hepatic stellate cell derivatives with IL-6. Representative Western blot analysis showing that the hepatic stellate cell derivatives LX-2 and GRX cells do not induce PLIN5 expression after treatment with IL-6 (10 ng/mL) for 24 h. In this analysis, Hep3B cells were used as controls and probing with GAPDH served as a control to demonstrate equal protein loading.
